# Routine Screening for Antibodies to Human Immunodeficiency Virus in the U.S. Armed Forces, Active and Reserve Components, January 2020–June 2025

**Published:** 2026-01-26

## Abstract

This report provides an update, through June 2025, of routine screening results for antibodies to the human immunodeficiency virus (HIV) among members of the U.S. military. The HIV-antibody seropositivity rates for active component service members from 2024 through mid-year 2025 were highest for the Navy (0.23 per 1,000 tested) and Marine Corps (0.22 per 1,000 tested), followed by the Army (0.17 per 1,000 tested), and lowest for the Air Force (0.13 per 1,000 tested) and Coast Guard (0.11 per 1,000 tested). Midyear HIV seropositivity rates, in comparison to 2024, increased for active component service members of the Army but decreased or remained stable for all other services, as of June 2025.

**What are the new findings?**

From January 2020 through June 2025, approximately 7 million U.S. military service members among the active component, reserve component, National Guard) were tested for antibodies to HIV, and 1,463 were identified as HIV-antibody-positive (seropositivity 0.21 per 1,000 tested). Of the 1,463 new infections identified during this period, only 40 (2.7%) were among female service members.

**What is the impact on readiness and force health protection?**

The HIV-antibody screening program remains an important element of U.S. force health protection, particularly for men under age 35 years, for all branches of service and service components. The measurement of military retention for HIV-positive service members reflects changes in U.S. Department of Defense policies that allow asymptomatic individuals with undetectable viral loads to serve without restrictions.

The U.S. Department of Defense (DOD) has conducted an active surveillance program for HIV since 1986. All service members of the active component, reserve component and National Guard are screened at specific points in time: prior to entry (all accessions must be HIV-negative prior to the start of service), before deployment or any change in status (e.g., change in component, between branches, or commissioning), and once every 2 years while a member of the U.S. military. ^1^ From 1990 through 2024, over 46 million tests for HIV antibodies were conducted to screen service members of the U.S. Armed Forces, resulting in the identification of 11,280 HIV new diagnoses (24.3 per 100,000 persons tested). While initial control efforts barred HIV-positive individuals from entering or serving in the military, leading to a precipitous drop in the rate of HIV diagnoses during the first decade of screening, the rate has remained stable for the last 2 decades. ^2^

Infection with HIV remains a disqualifying diagnosis for entry into U.S. military service; however, in June 2022, the DOD amended policies to prevent HIV-positive service members with an undetectable viral load from being discharged or separated solely on the basis of HIV status. ^1^ In addition, HIV-positive personnel are not non-deployable solely for a positive status, as decisions related to deployability should be made on a case-by-case basis, justified by a service member's ability to perform assigned duties. ^3^

Summaries of HIV seropositivity for members of the U.S. military have been published with *MSMR* since 1995. The current report summarizes numbers and trends of newly identified HIV-antibody seropositivity from January 1, 2020 through June 30, 2025 among military members of 5 services under the active and reserve components of the U.S. Armed Forces, in addition to the Army and Air Force National Guard.

## Methods

The surveillance population included all individuals eligible for HIV antibody screening from January 1, 2020 through June 30, 2025 while serving in the active or reserve components of the U.S. Army, Navy, Air Force, Marine Corps, or Coast Guard. Space Force service members were categorized as Air Force for this analysis. All individuals who were tested, and all initial detections of HIV antibodies, through U.S. military medical testing programs were ascertained from the Department of Defense Serum Repository (DODSR) specimens accessioned to the Defense Medical Surveillance System (DMSS).

An incident case of HIV-antibody seropositivity was defined as an individual with positive HIV test results on 2 different, serial specimens. Individuals who had just 1 positive result without a subsequent negative result were also defined as positive, to capture those who had yet to test positive for a second time. The total number of HIV-positive tests were acquired from DMSS to calculate seropositivity rates as a standardized methodology for all services.

Annual rates of HIV seropositivity among service members were calculated by dividing the number of incident cases of HIV-antibody seropositivity during each calendar year by the number of individuals tested at least once during the relevant calendar year. Rates were further stratified by service, component, and sex. Overall rates by age category were calculated for all services for the complete annual years 2020 through 2024.

## Results

**FIGURE 1.**
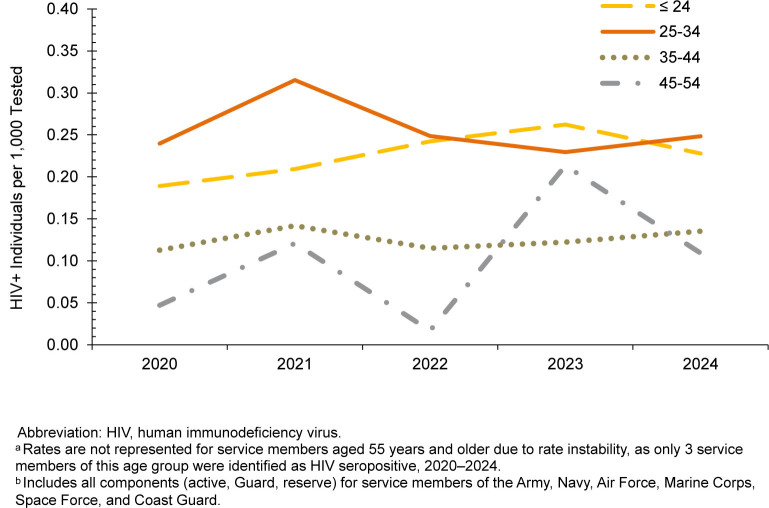
HIV Antibody Seropositivity Rates by Age ^a^ , U.S. Armed Forces ^b^ , 2020–2024

From January 2020 through June 2025, approximately 7 million service members (active component, Guard, reserve) were tested for antibodies to HIV, and 1,463 were identified as HIV-antibody-positive (seropositivity 0.21 per 1,000 tested) (data not shown). The male rate (0.26 per 1,000 tested) persisted above the female rate (0.03 per 1,000 tested) throughout the surveillance period, as only 40 women were identified as newly HIV-antibody-positive during this time. Age-specific HIV seropositivity rates are presented for complete annual years in **Figure 1** ; service members 25 to 34 years continually represented the highest age-specific rates from 2020 to 2024. In 2023, the seropositivity rate for service members ages 45-54 years increased to 0.21 per 1,000 tested, corresponding to an increase from 1 HIV seropositive cases identified in 2022 to 12 cases in 2023 (data not shown).

### U.S. Army, active component

**FIGURE 2.**
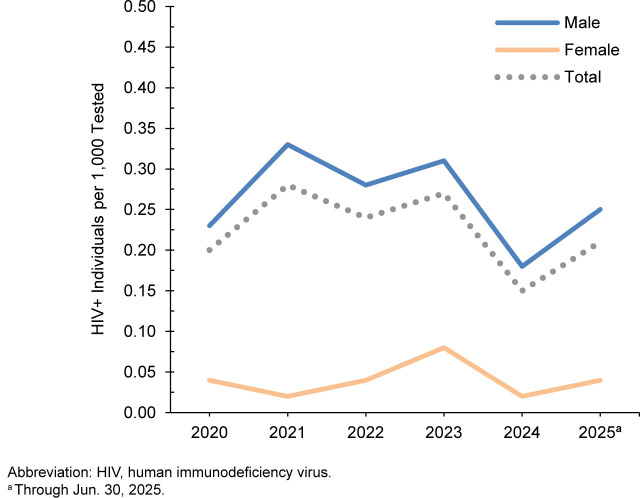
HIV Antibody Seropositivity Rates by Sex, Active Component, U.S. Army, January 2020–June 2025

From January 2024 through June 2025, a total of 445,309 U.S. Army active component soldiers were tested for HIV antibodies, and 77 were identified as HIV-antibody-positive (sero positivity 0.17 per 1,000 tested) **(Table 1)** . During the surveillance period, annual seropositivity rates fluctuated between a low of 0.15 per 1,000 tested in 2024 and a high of 0.28 per 1,000 tested in 2021 **(Table 1** , **Figure 2)** . Annual seropositivity rates for male active component soldiers were considerably higher than the seropositivity rates of female active component sol diers **(Figure 2)** . In 2024, 1 new HIV infec tion on average was detected among active component soldiers per 8,051 screening tests **(Table 1)** . Of the 389 active component soldiers diagnosed since 2020 with HIV infection, 242 (62.2%) were still in military service in 2025.

**TABLE 1.** New Diagnoses of HIV Infections, by Sex, U.S. Army, Active Component, January 2020–June 2025

Year	Total HIV Tests	Total Persons Tested	Male Tested	Female Tested	Total New HIV(+)	New HIV(+) Males	New HIV(+) Females	Overall Rate per 1,000 Tested	Male Rate per 1,000 Tested	Female Rate per 1,000 Tested	HIV(+) Still in Military Service in 2025
2020	398,322	322,343	269,973	52,370	65	63	2	0.20	0.23	0.04	26
2021	403,660	323,463	270,828	52,635	90	89	1	0.28	0.33	0.02	40
2022	373,983	306,689	256,691	49,998	74	72	2	0.24	0.28	0.04	46
2023	374,734	303,704	254,159	49,545	83	79	4	0.27	0.31	0.08	60
2024	378,383	303,701	251,934	51,767	47	46	1	0.15	0.18	0.02	40
2025 ^a^	151,416	141,608	116,781	24,827	30	29	1	0.21	0.25	0.04	30
Total	2,080,498	1,701,508	1,420,366	281,142	389	378	11	0.23	0.27	0.04	242

Abbreviation: HIV, human immunodeficiency virus.

a Through Jun. 30, 2025.

### Army National Guard

From January 2024 through June 2025, a total of 286,365 U.S. Army National Guard members were tested for HIV anti-bodies, and 102 soldiers were identified as HIV-antibody-positive (seropositivity 0.36 per 1,000 tested) **(Table 2)** . On average, 1 new HIV infection was detected in 2024 among Army National Guard soldiers per 3,309 screening tests. Of the 301 National Guard soldiers diagnosed since 2020 with HIV infection, 214 (71.1%) were still in service in 2025.

**TABLE 2.** New Diagnoses of HIV Infections, by Sex, U.S. Army National Guard, January 2020–June 2025

Year	Total HIV Tests	Total Persons Tested	Males Tested	Females Tested	Total New HIV(+)	New HIV(+) Males	New HIV(+) Females	Overall Rate per 1,000 Tested	Male Rate per 1,000 Tested	Female Rate per 1,000 Tested	HIV(+) Still in Military Service in 2025
2020	215,699	189,937	153,399	36,538	61	58	3	0.32	0.38	0.08	28
2021	218,060	190,121	154,006	36,115	50	48	2	0.26	0.31	0.06	28
2022	207,651	179,212	143,905	35,307	40	37	3	0.22	0.26	0.08	28
2023	214,471	186,821	149,597	37,224	48	47	1	0.26	0.31	0.03	37
2024	208,449	182,088	144,057	38,031	63	61	2	0.35	0.42	0.05	54
2025 ^a^	107,289	104,277	81,762	22,515	39	38	1	0.37	0.46	0.04	39
Total	1,171,619	1,032,456	826,726	205,730	301	289	12	0.29	0.35	0.06	214

Abbreviation: HIV, human immunodeficiency virus.

a Through Jun. 30, 2025.

### Army Reserve

From January 2024 through June 2025, a total of 127,024 U.S. Army Reserve members were tested for HIV antibodies, and 42 were identified as HIV-antibody-positive (seropositiv ity 0.33 per 1,000 tested) **(Table 3)** . During 2024, on average 1 new HIV infection was detected among Army reservists per 3,965 screening tests. Of the 153 Army reservists diagnosed since 2020 with HIV infection, 105 (68.6%) were still in service in 2025.

**TABLE 3.** New Diagnoses of HIV Infections, by Sex, U.S. Army Reserve, January 2020–June 2025

Year	Total HIV Tests	Total Persons Tested	Males Tested	Females Tested	Total New HIV(+)	New HIV(+) Males	New HIV(+) Females	Overall Rate per 1,000 Tested	Male Rate per 1,000 Tested	Female Rate per 1,000 Tested	HIV(+) Still in Military Service in 2025
2020	115,380	101,138	75,246	25,892	24	23	1	0.24	0.31	0.04	9
2021	119,108	101,434	75,562	25,872	29	29	0	0.29	0.38	0.00	17
2022	104,384	90,597	67,120	23,477	34	34	0	0.38	0.51	0.00	19
2023	79,437	69,082	50,640	18,442	24	23	1	0.35	0.45	0.05	18
2024	99,128	87,639	64,225	23,414	25	25	0	0.29	0.39	0.00	25
2025 ^a^	40,566	39,385	28,577	10,808	17	16	1	0.43	0.56	0.09	17
Total	558,003	489,275	361,370	127,905	153	150	3	0.31	0.42	0.02	105

Abbreviation: HIV, human immunodeficiency virus.

a Through Jun. 30, 2025.

### U.S. Navy, active component

**FIGURE 3.**
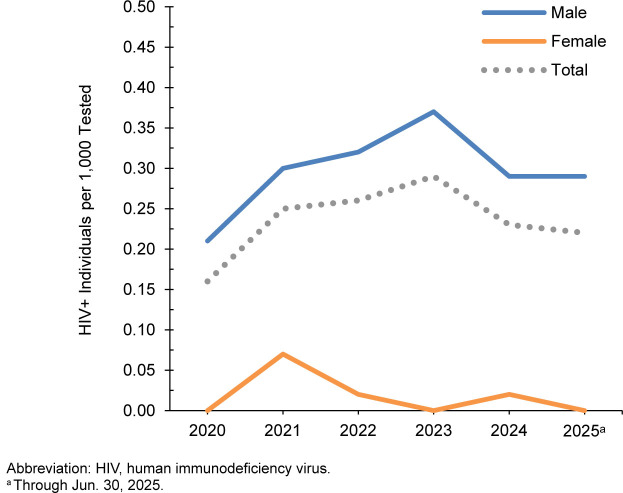
HIV Antibody Seropositivity Rates by Sex, Active Component, U.S. Navy, January 2020–June 2025

A total of 282,755 members of the U.S. Navy active compo nent were tested for HIV antibodies from January 2024 through June 2025, and 65 sailors were identified as HIV-antibody-positive (seropositivity 0.23 per 1,000 tested) **(Table 4)** . During the surveillance period, annual seropositivity rates fluctu ated between a low of 0.16 per 1,000 tested in 2020 and a high of 0.29 per 1,000 tested in 2023 **(Table 4** , **Figure 3)** . Annual seropositivity rates for male active component sailors were considerably higher than the seropositivity rates of female active component soldiers **(Figure 3)** . During 2024, on average, 1 new HIV infection was detected among active component sailors per 4,990 screening tests. Of the 256 active component sailors diagnosed since 2020 with HIV infection, 181 (70.7%) were still in service in 2025.

**TABLE 4.** New Diagnoses of HIV Infections, by Sex, U.S. Navy, Active Component, January 2020–June 2025

Year	Total HIV Tests	Total Persons Tested	Males Tested	Females Tested	Total New HIV(+)	New HIV(+) Males	New HIV(+) Females	Overall Rate per 1,000 Tested	Male Rate per 1,000 Tested	Female Rate per 1,000 Tested	HIV(+) Still in Military Service in 2025
2020	224,613	199,496	156,084	43,412	32	32	0	0.16	0.21	0.00	15
2021	242,438	215,079	168,997	46,082	54	51	3	0.25	0.30	0.07	30
2022	226,495	195,721	152,747	42,974	50	49	1	0.26	0.32	0.02	33
2023	223,122	191,930	149,570	42,360	55	55	0	0.29	0.37	0.00	47
2024	224,561	193,754	151,042	42,712	45	44	1	0.23	0.29	0.02	36
2025 ^a^	92,457	89,001	68,739	20,262	20	20	0	0.22	0.29	0.00	20
Total	1,233,686	1,084,981	847,179	237,802	256	251	5	0.24	0.30	0.02	181

Abbreviation: HIV, human immunodeficiency virus.

a Through Jun. 30, 2025.

### Navy Reserve

From January 2024 through June 2025, a total of 45,073 members of the U.S. Navy Reserve were tested for HIV antibodies, with 9 sailors identified as HIV-antibody-positive (seropositivity 0.20 per 1,000 tested) **(Table 5)** . On average, 1 new HIV infection was detected in 2024 among Navy reservists per 4,468 screening tests. Of the 33 reserve component sailors diagnosed since 2020 with HIV infection, 19 (57.6%) were still in service in 2025.

**TABLE 5.** New Diagnoses of HIV Infections, by Sex, U.S. Navy Reserve, January 2020–June 2025

Year	Total HIV Tests	Total persons Tested	Males Tested	Females Tested	Total New HIV(+)	New HIV(+) Males	New HIV(+) Females	Overall Rate per 1,000 Tested	Male Rate per 1,000 Tested	Female Rate per 1,000 Tested	HIV(+) Still in Military Service in 2025
2020	30,252	27,847	21,137	6,710	6	6	0	0.22	0.28	0.00	3
2021	36,499	33,185	25,041	8,144	11	9	2	0.33	0.36	0.25	3
2022	32,234	28,762	21,567	7,195	7	5	2	0.24	0.23	0.28	6
2023	32,650	29,757	22,144	7,613	0	0	0	0.00	0.00	0.00	0
2024	31,278	28,411	20,995	7,416	7	7	0	0.25	0.33	0.00	6
2025 ^a^	17,260	16,662	12,391	4,271	2	2	0	0.12	0.16	0.00	1
Total	180,173	164,624	123,275	41,349	33	29	4	0.20	0.24	0.10	19

Abbreviation: HIV, human immunodeficiency virus.

a Through Jun. 30, 2024.

### U.S. Air Force, active component

**FIGURE 4.**
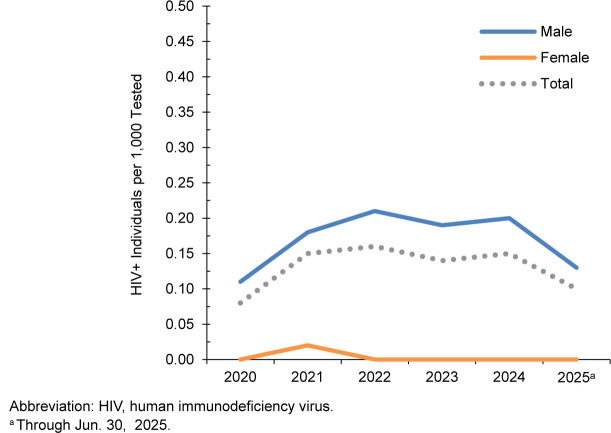
HIV Antibody Seropositivity Rates by Sex, Active Component, U.S. Air Force, January 2020–June 2025

From January 2024 through June 2025, a total of 274,169 active component members of the U.S. Air Force were tested for HIV antibodies, and 37 Air Force members were diagnosed with HIV infection (seropositivity 0.13 per 1,000 tested) **(Table 6)** . On average, 1 new HIV infection was detected in 2024 among active component Air Force members per 8,692 screening tests. Of the 143 active component Air Force members diagnosed since 2020 with HIV infection, 91 (63.6%) were still in service in 2025. During the surveillance period, seropositivity rates among male members ranged from a low of 0.08 per 1,000 tested in 2020 to a high of 0.16 per 1,000 tested in 2022 **(Figure 4)** .

**TABLE 6.** New Diagnoses of HIV Infections, U.S. Air Force, by Sex, Active Component, January 2020–June 2025

Year	Total HIV Tests	Total Persons Tested	Males Tested	Females Tested	Total New HIV(+)	New HIV(+) Males	New HIV(+) Females	Overall Rate per 1,000 Tested	Male Rate per 1,000 Tested	Female Rate per 1,000 Tested	HIV(+) Still in Military Service in 2025
2020	243,703	194,471	152,318	42,153	16	16	0	0.08	0.11	0.00	9
2021	256,751	208,332	162,305	46,027	31	30	1	0.15	0.18	0.02	14
2022	238,720	190,564	148,666	41,898	31	31	0	0.16	0.21	0.00	16
2023	254,519	193,510	151,182	42,328	28	28	0	0.14	0.19	0.00	19
2024	234,672	174,910	136,596	38,314	27	27	0	0.15	0.20	0.00	23
2025 ^a^	117,031	99,259	77,542	21,717	10	10	0	0.10	0.13	0.00	10
Total	1,345,396	1,061,046	828,609	232,437	143	142	1	0.13	0.17	0.00	91

Abbreviation: HIV, human immunodeficiency virus.

a Through Jun. 30, 2025.

### Air National Guard

From January 2024 through June 2025, a total of 85,121 members of the Air National Guard were tested for HIV anti-bodies, and 8 Air National Guard members were diagnosed with HIV infection (seropositivity 0.09 per 1,000 airmen tested) **(Table 7)** . During 2024, on average 1 new HIV infection was detected among Air National Guard members per 13,930 screening tests. Of the 32 Air National Guard members diagnosed since 2020 with HIV infection, 24 (75.0%) were still in service in 2025.

**TABLE 7.** New Diagnoses of HIV Infections, by Sex, U.S. Air National Guard, January 2020–June 2025

Year	Total HIV Tests	Total Persons Tested	Males Tested	Females Tested	Total New HIV(+)	New HIV(+) Males	New HIV(+) Females	Overall Rate per 1,000 Tested	Male Rate per 1,000 Tested	Female Rate per 1,000 Tested	HIV(+) Still in Military Service in 2025
2020	67,949	58,974	46,171	12,803	6	5	1	0.10	0.11	0.08	3
2021	68,112	60,311	47,168	13,143	8	8	0	0.13	0.17	0.00	5
2022	61,356	54,829	42,790	12,039	4	3	1	0.07	0.07	0.08	2
2023	70,837	56,978	44,647	12,331	6	6	0	0.11	0.13	0.00	6
2024	69,651	52,553	41,057	11,496	5	5	0	0.10	0.12	0.00	5
2025 ^a^	38,829	32,568	25,588	6,980	3	3	0	0.09	0.12	0.00	3
Total	376,734	316,213	247,421	68,792	32	30	2	0.10	0.12	0.03	24

Abbreviation: HIV, human immunodeficiency virus.

a Through Jun. 30, 2025.

### Air Force Reserve

From January 2024 through June 2025, a total of 51,770 members of the Air Force Reserve were tested for HIV antibodies, with 9 Air Force reservists diagnosed with HIV infection (seropositivity 0.17 per 1,000 tested) **(Table 8)** . On average, in 2024 1 new HIV infection was detected among Air Force reservists per 7,749 screening tests. Of the 38 Air Force reservists diagnosed since 2020 with HIV infection, 26 (68.4%) were still in service in 2025.

**TABLE 8.** New Diagnoses of HIV Infections, by Sex, U.S. Air Force Reserve, January 2020–June 2025

Year	Total HIV Tests	Total persons Tested	Males Tested	Females Tested	Total New HIV(+)	New HIV(+) Males	New HIV(+) Females	Overall Rate per 1,000 Tested	Male Rate per 1,000 Tested	Female Rate per 1,000 Tested	HIV(+) Still in Military Service in 2025
2020	38,943	33,947	24,604	9,343	6	6	0	0.18	0.24	0.00	3
2021	41,589	37,431	27,022	10,409	15	14	1	0.40	0.52	0.10	8
2022	37,274	33,460	24,185	9,275	4	4	0	0.12	0.17	0.00	4
2023	39,003	33,710	24,383	9,327	4	4	0	0.12	0.16	0.00	3
2024	38,743	31,854	22,645	9,209	5	5	0	0.16	0.22	0.00	4
2025 ^a^	22,173	19,916	14,405	5,511	4	4	0	0.20	0.28	0.00	4
Total	217,725	190,318	137,244	53,074	38	37	1	0.20	0.27	0.02	26

Abbreviation: HIV, human immunodeficiency virus.

a Through Jun. 30, 2025.

### U.S. Marine Corps, active component

**FIGURE 5.**
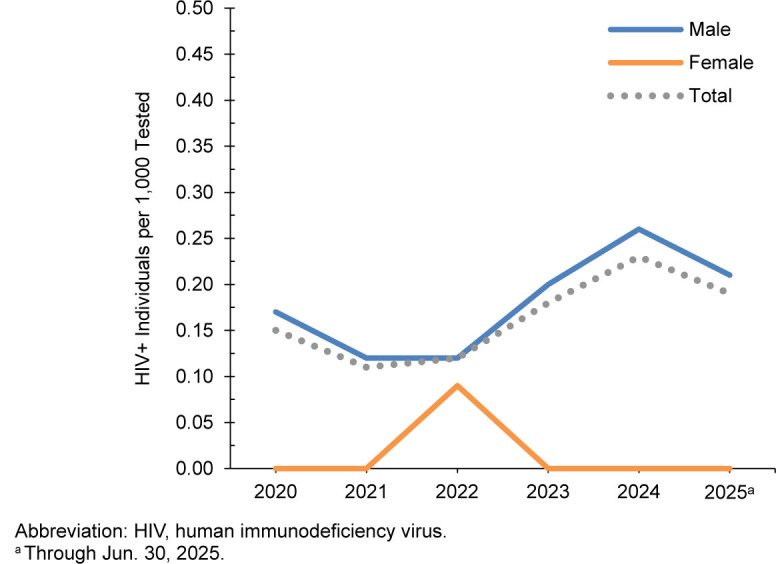
HIV Antibody Seropositivity Rates by Sex, Active Component, U.S. Marine Corps, January 2020–June 2025

From January 2024 through June 2025, a total of 154,093 active component members of the U.S. Marine Corps were tested for HIV antibodies, and 34 were identified as HIV-antibody-positive (seropositivity 0.22 per 1,000 tested) **(Table 9)** . Annual seropositivity rates rose from a low of 0.11 per 1,000 tested in 2021 to a high of 0.23 per 1,000 tested in 2024 **(Table 9** , **Figure 5)** . In 2024, on average, 1 new HIV infection per 5,031 screening tests was detected among active component marines. Of the 100 active component marines diagnosed since 2020 with HIV infection, 58 (58.0%) were still in service in 2025.

**TABLE 9.** New Diagnoses of HIV Infections, by Sex, U.S. Marine Corps, Active Component, January 2020–June 2025

Year	Total HIV Tests	Total Persons Tested	Males Tested	Females Tested	Total New HIV(+)	New HIV(+) Males	New HIV(+) Females	Overall Rate per 1,000 Tested	Male Rate per 1,000 Tested	Female Rate per 1,000 Tested	HIV(+) Still in Military Service in 2025
2020	140,663	123,760	112,634	11,126	19	19	0	0.15	0.17	0.00	5
2021	148,034	129,763	117,790	11,973	14	14	0	0.11	0.12	0.00	4
2022	129,465	112,850	101,857	10,993	13	12	1	0.12	0.12	0.09	6
2023	129,606	112,505	101,138	11,367	20	20	0	0.18	0.20	0.00	14
2024	125,782	107,279	96,195	11,084	25	25	0	0.23	0.26	0.00	20
2025 ^a^	48,642	46,814	42,121	4,693	9	9	0	0.19	0.21	0.00	9
Total	722,192	632,971	571,735	61,236	100	99	1	0.16	0.17	0.02	58

Abbreviation: HIV, human immunodeficiency virus.

a Through Jun. 30, 2025.

### Marine Corps Reserve

From January 2024 through June 2025, a total of 28,972 Marine Corps Reserve members were tested for antibodies to HIV, and 2 reservists were identified as HIV-antibody-positive (seropositivity 0.07 per 1,000 tested) **(Table 10)** . During 2024, on average, 1 new HIV infection was detected among Marine Corps reservists per 10,730 screening tests. Of the 12 active component marine reservists diagnosed since 2020 with HIV infection, 5 (41.7%) were still in service in 2025.

**TABLE 10.** New Diagnoses of HIV Infections, by Sex, U.S. Marine Corps Reserve, January 2020–June 2025

Year	Total HIV Tests	Total Persons Tested	Males Tested	Females Tested	Total New HIV(+)	New HIV(+) Males	New HIV(+) Females	Overall Rate per 1,000 Tested	Male Rate per 1,000 Tested	Female Rate per 1,000 Tested	HIV(+) Still in Military Service in 2025
2020	19,371	17,874	17,141	733	2	2	0	0.11	0.12	0.00	1
2021	26,095	22,700	21,840	860	6	6	0	0.26	0.27	0.00	2
2022	19,963	17,747	17,024	723	2	2	0	0.11	0.12	0.00	0
2023	21,580	19,194	18,310	884	0	0	0	0.00	0.00	0.00	0
2024	21,460	18,533	17,626	907	2	2	0	0.11	0.11	0.00	2
2025 ^a^	10,660	10,439	9,872	567	0	0	0	0.00	0.00	0.00	0
Total	119,129	106,487	101,813	4,674	12	12	0	0.11	0.12	0.00	5

Abbreviation: HIV, human immunodeficiency virus.

a Through Jun. 30, 2025.

### U.S. Coast Guard, active component

From January 2024 through June 2025, a total of 28,188 active component members of the U.S. Coast Guard were tested for antibodies to HIV, and 3 were identified as HIV-antibody-positive (seropositivity 0.11 per 1,000 tested) **(Table 11)** . During 2024, on average, 1 new HIV infection was detected among active component members of the U.S Coast Guard per 9,920 screening tests. Of the 5 active component Coast Guard service members diagnosed since 2020 with HIV infection, all 5 were still in service in 2025.

**TABLE 11.** New Diagnoses of HIV Infections, by Sex, U.S. Coast Guard, Active Component, January 2020–June 2025

Year	Total HIV Tests	Total Persons Tested	Males Tested	Females Tested	Total New HIV(+)	New HIV(+) Males	New HIV(+) Females	Overall Rate per 1,000 Tested	Male Rate per 1,000 Tested	Female Rate per 1,000 Tested	HIV(+) Still in Military Service in 2025
2020	17,269	16,748	14,133	2,615	1	1	0	0.06	0.07	0.00	1
2021	20,463	19,800	16,632	3,168	1	1	0	0.05	0.06	0.00	1
2022	19,578	18,934	15,930	3,004	0	0	0	0.00	0.00	0.00	0
2023	19,290	18,495	15,480	3,015	0	0	0	0.00	0.00	0.00	0
2024	19,839	18,890	15,786	3,104	2	2	0	0.11	0.13	0.00	2
2025 ^a^	9,497	9,298	7,752	1,546	1	1	0	0.11	0.13	0.00	1
Total	105,936	102,165	85,713	16,452	5	5	0	0.05	0.06	0.00	5

Abbreviation: HIV, human immunodeficiency virus.

a Through Jun. 30, 2025.

### Coast Guard Reserve

From January 2024 through June 2025, a total of 5,307 U.S. Coast Guard Reserve members were tested for HIV antibodies, with none identified as HIV-antibody-positive **(Table 12)** .

**TABLE 12.** New Diagnoses of HIV Infections, by Sex, U.S. Coast Guard Reserve, January 2020–June 2025

Year	Total HIV Tests	Total Persons Tested	Males Tested	Females Tested	Total New HIV(+)	New HIV(+) Males	New HIV(+) Females	Overall Rate per 1,000 Tested	Male Rate per 1,000 Tested	Female Rate per 1,000 Tested	HIV(+) Still in Military Service in 2025
2020	2,846	2,756	2,284	472	1	1	0	0.36	0.44	0.00	1
2021	3,233	3,027	2,514	513	0	0	0	0.00	0.00	0.00	0
2022	2,918	2,826	2,332	494	0	0	0	0.00	0.00	0.00	0
2023	2,907	2,788	2,275	513	0	0	0	0.00	0.00	0.00	0
2024	3,218	3,059	2,530	529	0	0	0	0.00	0.00	0.00	0
2025 ^a^	2,268	2,248	1,865	383	0	0	0	0.00	0.00	0.00	0
Total	17,390	16,704	13,800	2,904	1	1	0	0.06	0.07	0.00	1

Abbreviation: HIV, human immunodeficiency virus.

a Through Jun. 30, 2025.

## Discussion

The most current seropositivity rate (0.21 per 1,000 tested) reported for January 1, 2024 through June 30, 2025 remains consistent with the seropositivity rate reported in the prior annual report (0.22 per 1,000 tested from January 1, 2023 to June 30, 2025). ^4^ The U.S. military has conducted routine screening for antibodies to HIV among all civilian applicants for service and all service members for more than 30 years. ^5 - 8^ In 1995, the U.S. Army tested approximately 1.1 million specimens annually, demonstrating an economically efficient, large-scale model for HIV testing. ^9^ The first *MSMR* article to publish results from HIV screening programs indicates that antibody seropositivity rates in 1994 for the Army active duty (0.19 per 1,000 soldiers) and reserve component (0.23 per 1,000 soldiers) remain comparable to rates presented in 2025. ^10^

A review of archived surveillance data also reflects improved retention of HIV-positive service members, in alignment with recent DOD policy that recognizes significant advances in the diagnosis, prevention, and treatment of the disease. From 1990 to 1994, a total of 889 active and reserve component soldiers were diagnosed with HIV-1 infection. By 1995, only 234 (26.0%) were still in service. ^10^ Today, a comparative retention figure for active component Army service members has increased to 66.4%.

The 2022-2025 National HIV/AIDS strategy identifies youth ages 13-24 years as a priority population, based on increased risk for HIV transmission. ^11^ While the seropositivity results presented in this report do partially represent this priority population, as over 43% of all new HIV infections were identified in service members younger than age 25 years, these results should not be generalized to the U.S. population. Data from HIV screening in U.S. military populations are based on a negative test prior to entry, as well as voluntary service. Previous *MSMR* reports presented HIV screening results for civilian applicants to the military service; however, those data are no longer available in the Defense Medical Surveillance System (DMSS), as the U.S. Military Entrance Processing Command stopped reporting data to the DMSS at the end of calendar year 2020. Thus, the data presented in this report reflect service members who had a negative HIV test upon entry to military service, followed by a positive test during uniformed service.

Routine screening of civilian applicants for service and periodic testing of all active and reserve component members have been fundamental components of the military's HIV control and clinical management efforts. ^12^ The most current HIV annual seropositivity rates indicate the HIV-antibody screening program remains an important element of force health protection, particularly for men younger than age 35 years, for all branches of service and components of the U.S. Armed Forces.
